# Comparison of real-time PCR and hemagglutination assay for quantitation of human polyomavirus JC

**DOI:** 10.1186/1743-422X-3-3

**Published:** 2006-01-09

**Authors:** Moti L Chapagain, Taylor Nguyen, Thomas Bui, Saguna Verma, Vivek R Nerurkar

**Affiliations:** 1 Retrovirology Research Laboratory, Department of Tropical Medicine, Medical Microbiology and Pharmacology, Asia-Pacific Institute of Tropical Medicine and Infectious Diseases, John A. Burns of School of Medicine, University of Hawaii, 651 Ilalo Street, BSB 325AA, Honolulu, Hawaii 96813, USA

## Abstract

Human polyomavirus JC (JCV), the etiological agent of the disease progressive multifocal leukoencephalopathy (PML) affects immunocompromised patients particularly patients with AIDS. *In vitro *studies of JCV infection are hampered by the lack of sensitive JCV quantitation tests. Although the hemagglutination (HA) assay has been routinely employed for *in vitro *quantitation of JCV, its sensitivity is severely limited. We have employed a real-time PCR assay which compares favorably with the HA assay for the *in vitro *quantitation of JCV. JCV(Mad1), propagated in primary human fetal glial (PHFG) cells in two independent laboratories, was purified and quantitated by the HA assay. Both batches of purified JCV(Mad1) were then serially diluted in Dulbecco's Modified Eagle's Medium to obtain HA titers ranging from 64 to 0.001 HA units (HAU) per 100 μL of virus suspension. DNA was extracted from 100 μL of virus suspension and eluted in 50 μL of buffer, and DNA amplification and quantitation were performed in the Bio-Rad iCycler iQ Multicolor Real-Time PCR Detection System using T-antigen as the target gene. Real-time PCR for quantitation of JCV was sensitive and consistently detected 1.8 × 10^1 ^copies of JCV DNA, and as low as 0.001 HAU equivalent of JCV. Moreover, there was a strong linear correlation between the HA assay and the DNA copy number of JCV(Mad1). The intra-run and inter-run coefficients of variation for the JCV standard curve were 0.06% to 4.8% and 2.6% to 5.2%, respectively. Based on these data, real-time PCR can replace the less-sensitive HA assay for the reliable detection, quantitation and monitoring of *in vitro *JCV replication.

## Findings

Human polyomavirus JC (JCV), a small, non-enveloped virus with a closed circular double-stranded-DNA genome, is ubiquitous in nature with a seroprevalence of up to 80% among geographically isolated populations [1-3]. JCV was first isolated in 1971 from the brain of a patient with progressive mulifocal leukoencephalopathy (PML) [4]. Most primary JCV infections occur during childhood [3], and are subclinical. However, JCV remains latent for life and may cause a fatal demyelinating disease, PML, among immunocompromised patients [5]. PML remains a frequent and life-threatening complication of HIV infection and about 3–5% of AIDS patients develop this disease [6,7]. Factors influencing PML pathogenesis, including modes of JCV transmission, its dissemination from site(s) of initial infection, the mechanism(s) of JCV reactivation, cellular susceptibility, trafficking across the blood-brain-barrier and lytic infection of oligodendrocytes, are still unclear. Efforts to understand the pathogenesis of PML have been hampered by the lack of standard methods for JCV detection and quantitation.

JCV major capsid protein, VP-1, is responsible for red blood cell (RBC) agglutination and traditionally, hemagglutination (HA) and HA inhibition (HAI) assays have been employed for quantitation of JCV [8-14]. However, the HA assay is poorly sensitive and the *in vitro *quantitation of JC viral load by HA is often impossible. Moreover HA assay cannot be efficiently employed to study JCV replication in various experimental settings including, experiments employing microtiter and transwell plates. Semi-quantitative polymerase chain reaction (PCR) and quantitative real-time PCR have been recently developed and employed for the detection and quantitation of JCV [15-19]. In clinical specimens, real time-PCR has proven to be an important method for monitoring JCV viral load, [15,20] and is a reliable marker for PML prognosis [21]. However, it is unclear how changes in viral DNA levels are correlated with the virion levels/viral load and no effort has been made to standardize the copy-number equivalent of JCV required in *in vitro *JCV replication-related experiments. To further our knowledge of PML pathogenesis, uniformly controlled detection and quantitation techniques are essential for *in vitro *monitoring of JCV replication. In this study, we investigated the relationship between real-time PCR and HA assays for the determination of JC viral load.

JCV(Mad1) was propagated in primary human fetal glial (PHFG) cells and purified in Dr. Duard Walker's laboratory (I) [4,22,23] or at the Retrovirology Research Laboratory (RRL) (II), University of Hawaii [13,24]. PHFG cells, infected with JCV(Mad1) at RRL were harvested on day 30 post infection, subjected to three freeze-thaw cycles, disrupted by sonication at 100 watt for 1 min on ice using an Autotune series high-intensity ultrasonic processor, and incubated with 0.25% deoxycholic acid at 37°C for 1 hr. Cellular debris was removed by centrifugation at 5,000 rpm (1,960 × g) for 30 min and the supernatant was layered on 30% sucrose (w/w) and centrifuged at 35,000 rpm in a Beckman SW 55Ti rotor using a Beckman LE-80K ultracentrifuge (Beckman Coulter, Inc., Fullerton, CA). The supernatant was decanted and the pellet was suspended in Dulbecco's Modified Eagle's Medium (DMEM) and stored at -80°C. JCV titers were determined by the HA assay as described elsewhere [3,10]. Briefly, human type O blood was centrifuged at 2,500 rpm for 10 min at 4°C. RBC were then washed twice and suspended in Alsever's buffer (20 mM sodium citrate, 72 mM NaCl, 100 mM glucose, pH 6.5 adjusted with acetic acid) at a final concentration of 0.5%. Serial two-fold dilutions of virus suspensions were prepared in Alsever's buffer and 50 μL of viral suspension and an equal volume of RBC were added into each well of a 96-well "U" bottom microtiter plate and incubated at 4°C for 3–6 hr. The HA titer was the reciprocal of the final dilution of virus suspension that agglutinated RBC. The end point dilution was considered 1 hemagglutination unit (HAU).

Both batches of JCV(Mad1) (I and II) were serially diluted two-fold (64 HA to 1 HA) and then 10-fold (1 HA to 0.001 HA) in DMEM, to obtain HA titers ranging from 64 HAU to 0.001 HAU per 100 μL of the suspension. DNA was extracted from 100 μL of the above suspension containing known HAU of JCV using the QIAprep^® ^Spin Miniprep kit (Cat No. 27106), according to the manufacturer's instructions and DNA was eluted in 50 μL of elution buffer [25]. JCV DNA amplification and quantitation were performed in the Bio-Rad iCycler iQ™ Multicolor Real-Time PCR Detection System using 2 μL of 1:10 diluted template DNA, Bio-Rad 2X iQ™ SYBER^® ^Green supermix and 12.5 pmol each of forward and reverse primers specific for the JCV T-antigen gene [JCT-1 (Forward: 5' AGA GTG TTG GGA TCC TGT GTT TT 3'; JCT-2 (Reverse) 5' GAG AAG TGG GAT GAA GAC CTG TTT 3'] (GeneBank Accession No. J02226) [15] in a final reaction volume of 20 μL. Thermal cycling was initiated with a first denaturation step of 10 min at 95°C, followed by 40 cycles of 95°C for 10 sec and 60°C for 15 sec and the amplification fluorescence was read at 60°C at the end of the cycle. Real-time PCR amplification data were analyzed using the Bio-Rad iCycler iQ™ Multicolor Real-Time PCR Optical System Software Version 3.1. A standard curve for the quantitation of JCV was constructed using serial dilutions of the linearized JCV(Mad1) plasmid. The dynamic range of detection was determined by preparing 10-fold serial dilutions of JCV plasmid in the range of 10 pg to 1 fg, that represented 1.8 × 10^5 ^to 1.8 × 10^1 ^copies of JCV DNA, respectively. All experiments were done twice and samples were run in triplicate each time. Copies of JCV DNA in experimental samples were calculated from the standard curve and were expressed as copies of viral DNA per 100 μL of virus suspension. The reliability of real-time PCR was defined by calculating coefficients of variation of Ct values of replicates of standard curve dilutions [21,26].

**Figure 1:**
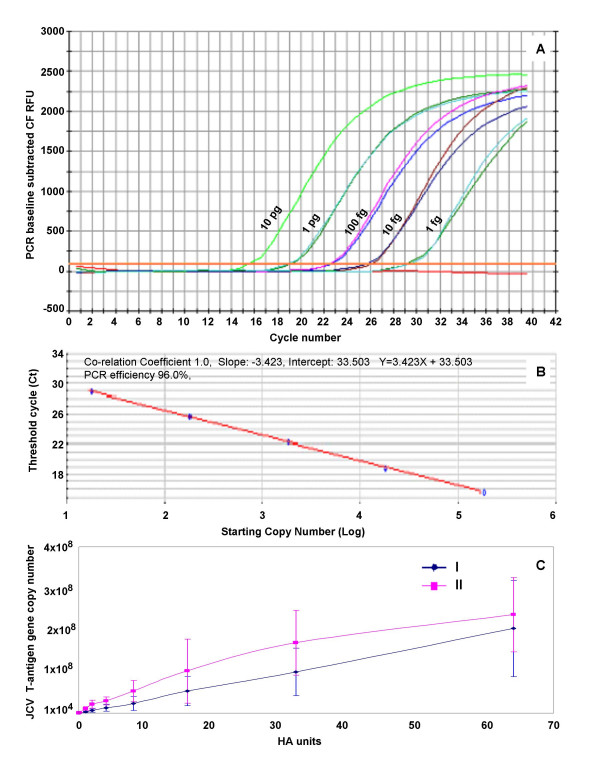
**A – C: Analysis of HA and quantitative real-time PCR data employed for the determination of JC viral load. **JCV (Mad1), propagated in Dr. Walker's laboratory (I) or propagated at the RRL (II) was serially diluted with DMEM to make 100 μl of viral suspension containing 64 HAU to 0.001 HAU of JCV. DNA was extracted using a QIAprep Miniprep Spin Kit and eluted in 50 μl of the elution buffer. Two μl of the 1:10 diluted template DNA was used for PCR in a final volume of 20 μl of PCR mixture. Copies of JCV(Mad1) T antigen gene were calculated from the standard curve and were expressed as copies of viral DNA per 100 μl of suspension. **A**) Amplification plots of relative fluorescence units (RFU) vs. cycle number of the JCV T antigen gene in known amounts of JCV plasmid DNA, ranging from 10 pg to 1 fg in decreasing 1:10 serial dilutions. **B**) Standard curve plot of the log of plasmid copy number against cycle threshold (Ct), where Ct is defined as the first cycle in which amplification signal is detected over mean baseline signal. The slope was -3.32 and r^2 ^= 1.00. **C**) The data representative of two independent experiments with samples tested in triplicate for each run of real-time PCR. Standard error bars are represented as standard deviation. The slopes were Y = 3 × 10^6^X - 350714 and Y = 4 × 10^6^X + 1 × 10^7 ^and the r^2 ^were 1.0 and 0.95 for JCV(Mad1) virus stocks I and II, respectively.

We first examined the fidelity of real-time quantitative PCR in detecting and quantitating JCV T-antigen gene sequences from clinical specimens. Quantitative real-time PCR proved to be an appropriate technique for detection of JCV DNA. The JCV T-antigen gene standard curve was highly reproducible and precise (Fig 1B). The intra-run and inter-run coefficients of variation for the standard curve varied from 0.06% to 4.8% and 2.6% to 5.2%, respectively, and thus appeared to be consistent for detecting and quantitating the JCV genome copy numbers. Moreover, re-extraction of DNA from 100 μL of JCV suspension, with known HAU, yielded less than a two-fold variation in JCV DNA copy numbers. Our JCV assay demonstrated a wide linear range and was able to detect as low as 1.8 × 10^1 ^copies of JCV DNA present in the template (Fig 1A) and 0.001 HAU equivalent of JCV in 100 μL of the virus suspension (data not shown). Thus real-time PCR was at least 1,000-fold more sensitive than the traditional HA assay in detecting JCV.

There was a strong linear correlation between the HA assay and the DNA copy number of JCV(Mad1) either propagated by Dr. Walker (I, r^2 ^= 1.0) or propagated at the RRL (II, r^2 ^= 0.95) (Fig 1C). JCV(Mad1) obtained from two different sources yielded very similar DNA T-antigen gene copy numbers from samples containing the same HAU of JCV. However, the difference was more pronounced when the suspension contained less than 4 HAU of JCV. This difference might have resulted from the presence of different proportions of either the empty virions or naked DNA. The ratio of empty virions and naked JCV DNA present in virus suspension may be affected by several factors including the virus strain, cell type used to propagate JCV, virus isolation procedures as well as time of harvesting the infected cells. Moreover, the less than two-fold difference in JCV copy numbers in two sources of virus may simply be the result of the less sensitive HA assay. The HA assay produced similar results when the virions in any two samples differed by less than two-fold.

Our data demonstrate that real-time PCR is a sensitive and reliable method for *in vitro *quantitation of JCV. *In vitro *measurement of JCV DNA levels was highly reproducible over a large dynamic range, and real-time PCR was more reliable than the HA assay for *in vitro *calculation of initial virus inoculum and replicated virus. In the future, quantitative real-time PCR can be employed to study *in vitro *efficacy of several potential therapeutic agents against JCV as well as to quantitate JCV trafficking across the blood-brain-barrier using an *in vitro *model. Real-time PCR detects as low as 0.001 HAU equivalent of JCV, eliminates the variability traditionally associated with the HA assay, and thus could reliably replace the HA assay employed for JCV replication in *in-vitro *pathogenesis studies.

## Competing interests

The author(s) declare that they have no competing interests.

## Authors' contributions

Design and conception of study (VRN, MLC); development of quantitative real-time PCR method (SV, TN); virus culture (TB, MLC); virus purification, DNA extraction, HA assay (MLC, VRN); manuscript preparation (MLC, VRN,). All authors read and approved the final manuscript.

## References

[B1] Rollison DE, Helzlsouer KJ, Alberg AJ, Hoffman S, Hou J, Daniel R, Shah KV, Major EO (2003). Serum antibodies to JC virus, BK virus, simian virus 40, and the risk of incident adult astrocytic brain tumors. Cancer Epidemiol Biomarkers Prev.

[B2] Kim HS, Henson JW, Frisque RJ, Khalili K and Stoner GL (2001). Transcription and replication in the human polyomaviruses. Human Polyomaviruses.

[B3] Padgett BL, Walker DL (1973). Prevalence of antibodies in human sera against JC virus, an isolate from a case of progressive multifocal leukoencephalopathy. J Infect Dis.

[B4] Padgett BL, Walker DL, ZuRhein GM, Eckroade RJ, Dessel BH (1971). Cultivation of papova-like virus from human brain with progressive multifocal leucoencephalopathy. Lancet.

[B5] Padgett BL, Walker DL, ZuRhein GM, Hodach AE, Chou SM (1976). JC Papovavirus in progressive multifocal leukoencephalopathy. J Infect Dis.

[B6] Koralnik IJ (2004). New insights into progressive multifocal leukoencephalopathy. Curr Opin Neurol.

[B7] Berger JR (2003). Progressive multifocal leukoencephalopathy in acquired immunodeficiency syndrome: explaining the high incidence and disproportionate frequency of the illness relative to other immunosuppressive conditions. J Neurovirol.

[B8] Seth P, Diaz F, Tao-Cheng JH, Major EO (2004). JC virus induces nonapoptotic cell death of human central nervous system progenitor cell-derived astrocytes. J Virol.

[B9] Hara K, Sugimoto C, Kitamura T, Aoki N, Taguchi F, Yogo Y (1998). Archetype JC virus efficiently replicates in COS-7 cells, simian cells constitutively expressing simian virus 40 T antigen. J Virol.

[B10] Neel JV, Major EO, Awa AA, Glover T, Burgess A, Traub R, Curfman B, Satoh C (1996). Hypothesis: "Rogue cell"-type chromosomal damage in lymphocytes is associated with infection with the JC human polyoma virus and has implications for oncopenesis. Proc Natl Acad Sci U S A.

[B11] Knowles WA, Luxton RW, Hand JF, Gardner SD, Brown DW (1995). The JC virus antibody response in serum and cerebrospinal fluid in progressive multifocal leucoencephalopathy. Clin Diagn Virol.

[B12] Knowles WA, Pipkin P, Andrews N, Vyse A, Minor P, Brown DW, Miller E (2003). Population-based study of antibody to the human polyomaviruses BKV and JCV and the simian polyomavirus SV40. J Med Virol.

[B13] Akatani K, Imai M, Kimura M, Nagashima K, Ikegami N (1994). Propagation of JC virus in human neuroblastoma cell line IMR-32. J Med Virol.

[B14] Hou J, Major EO (1998). The efficacy of nucleoside analogs against JC virus multiplication in a persistently infected human fetal brain cell line. J Neurovirol.

[B15] Ryschkewitsch C, Jensen P, Hou J, Fahle G, Fischer S, Major EO (2004). Comparison of PCR-southern hybridization and quantitative real-time PCR for the detection of JC and BK viral nucleotide sequences in urine and cerebrospinal fluid. J Virol Methods.

[B16] Whiley DM, Mackay IM, Sloots TP (2001). Detection and differentiation of human polyomaviruses JC and BK by LightCycler PCR. J Clin Microbiol.

[B17] Beck RC, Kohn DJ, Tuohy MJ, Prayson RA, Yen-Lieberman B, Procop GW (2004). Detection of polyoma virus in brain tissue of patients with progressive multifocal leukoencephalopathy by real-time PCR and pyrosequencing. Diagn Mol Pathol.

[B18] Watzinger F, Suda M, Preuner S, Baumgartinger R, Ebner K, Baskova L, Niesters HG, Lawitschka A, Lion T (2004). Real-time quantitative PCR assays for detection and monitoring of pathogenic human viruses in immunosuppressed pediatric patients. J Clin Microbiol.

[B19] Verma S, Ziegler K, Ananthula P, Co JKG, Frisque RJ, Yanagihara R, Nerurkar VR (2005). JC virus induces altered patterns of cellular gene expression: Interferon-inducible genes as major transcriptional targets. Virology.

[B20] Rollison DE, Utaipat U, Ryschkewitsch C, Hou J, Goldthwaite P, Daniel R, Helzlsouer KJ, Burger PC, Shah KV, Major EO (2005). Investigation of human brain tumors for the presence of polyomavirus genome sequences by two independent laboratories. Int J Cancer.

[B21] Bossolasco S, Calori G, Moretti F, Boschini A, Bertelli D, Mena M, Gerevini S, Bestetti A, Pedale R, Sala S, Lazzarin A, Cinque P (2005). Prognostic significance of JC virus DNA levels in cerebrospinal fluid of patients with HIV-associated progressive multifocal leukoencephalopathy. Clin Infect Dis.

[B22] Padgett BL, Rogers CM, Walker DL (1977). JC virus, a human polyomavirus associated with progressive multifocal leukoencephalopathy: additional biological characteristics and antigenic relationships. Infect Immun.

[B23] Osborn JE, Robertson SM, Padgett BL, Walker DL, Weisblum B (1976). Comparison of JC and BK human papovaviruses with simian virus 40: DNA homology studies. J Virol.

[B24] Liu CK, Hope AP, Atwood WJ (1998). The human polyomavirus, JCV, does not share receptor specificity with SV40 on human glial cells. J Neurovirol.

[B25] Ziegler K, Bui T, Frisque RJ, Grandinetti A, Nerurkar VR (2004). A rapid in vitro polyomavirus DNA replication assay. J Virol Methods.

[B26] Reed GF, Lynn F, Meade BD (2002). Use of coefficient of variation in assessing variability of quantitative assays. Clin Diagn Lab Immunol.

